# Supplementary material for: Correlating global trends in COVID-19 cases with online symptom checker self-assessments
Source: PLoS One. 2023 Feb 10;18(2):e0281709. doi: 10.1371/journal.pone.0281709 (PMC9917242; doi:10.1371/journal.pone.0281709)
Supplement: S1 Table — (DOCX) [file pone.0281709.s001.docx]

S1 Table: QOL Metrics, Correlations, and Shifted Correlations

| Country | Internet for Health Percent | Gini | Self Reported Health | Life Satisfaction | Correlation | Max Lagged Correlation | Delta of Max Lagged Correlation |
| --- | --- | --- | --- | --- | --- | --- | --- |
| Australia | 19.71 | 0.325 | 85 | 7.3 | -0.054 | -0.002 | -19 |
| Austria | 16.03 | 0.280 | 70 | 7.0 | 0.653 | 0.700 | 7 |
| Belgium | 19.14 | 0.258 | 75 | 6.9 | 0.401 | 0.434 | 12 |
| Canada | 37.18 | 0.301 | 88 | 7.3 | 0.280 | 0.329 | -1 |
| Switzerland | 46.10 | 0.299 | 80 | 7.5 | 0.215 | 0.273 | 12 |
| Czechia | 3.47 | 0.249 | 61 | 6.6 | 0.644 | 0.724 | 29 |
| Germany | 34.23 | 0.289 | 65 | 7.0 | 0.733 | 0.733 | 0 |
| Spain | 12.75 | 0.330 | 72 | 6.4 | 0.181 | 0.279 | -12 |
| France | 12.97 | 0.301 | 68 | 6.4 | 0.585 | 0.642 | -4 |
| United Kingdom | 25.46 | 0.366 | 70 | 6.7 | 0.488 | 0.514 | 6 |
| Greece | 2.16 | 0.306 | 74 | 5.2 | 0.835 | 0.852 | 5 |
| Hungary | 9.61 | 0.289 | 56 | 5.3 | 0.480 | 0.519 | 5 |
| Ireland | 10.48 | 0.292 | 82 | 7.0 | 0.011 | 0.103 | -16 |
| Italy | 8.73 | 0.330 | 66 | 5.9 | 0.461 | 0.510 | 13 |
| Netherlands | 40.72 | 0.285 | 76 | 7.4 | 0.313 | 0.367 | 27 |
| Sweden | 23.21 | 0.280 | 80 | 7.3 | -0.014 | 0.157 | -27 |
| Turkey | 3.12 | 0.397 | 66 | 5.5 | 0.134 | 0.404 | -24 |
| United States | 7.57 | 0.390 | 88 | 6.9 | -0.187 | -0.150 | -14 |
