# Supplementary material for: Correlating global trends in COVID-19 cases with online symptom checker self-assessments
Source: PLoS One. 2023 Feb 10;18(2):e0281709. doi: 10.1371/journal.pone.0281709 (PMC9917242; doi:10.1371/journal.pone.0281709)
Supplement: Attachment — Submitted filename: Rebuttal_Letter_PLOS_ONE.pdf [file pone.0281709.s002.pdf]

Dear Reviewers,

On behalf of all the authors of the paper, we would like to thank you for the in-depth review. The suggested changes have been mostly implemented after much careful consideration. In the following sections, we describe in detail how each comment was addressed:

> ***Reviewer 1:***
> *1- in the method, the normal or not normal distribution of the variables are required to be checked by test such as Kolmogorov–Smirnov (k-s) test and then the PCC to be used, Spearman correlation coefficient might be the suitable option if pvalue of K-S test >0.05. We need to check the data distribution and then the test can be chosen.*

As suggested, we tested the normality of each time series via the Kolmogorov-Smirnov test. We failed to reject the null hypothesis for most time series, the KS statistic indicating that they are non-normal (p-value > 0.05). Given this, we replaced the analyses based on the Pearson correlation coefficients throughout with the Spearman rank correlation coefficients. Previous observed trends and conclusions drawn from them were, in general, unaffected by this change.

> ***Reviewer 1:***
> *2- There is the lack of section to introduce the available data before analysis. Both variables according to the time and geography need to be summarized using descriptive indexes and the after having insight regarding the data , we need to progress for data analysis.*

We improved our Data section by expanding the description of the time frame, while also providing the count of the countries and the number of days in total. The list of countries included in this study is also now given in full.

> *Only countries with consecutive data, that being no days without a single high-risk assessment given, between 7th of April and 1st of November 2020 were included in the further analysis. This results in a dataset of 18 countries with 208 days in each. The respective countries are: Australia, Austria, Belgium, Canada, Czech Republic, France, Germany, Greece, Hungary, Ireland, Italy, Netherlands, Spain, Sweden, Switzerland, Turkey, United Kingdom, United States. Within the text, we refer to this data as 'Symptoma risk assessments'.*

Alongside the above text, we also inserted additional instances of reference to our previous work which give an overview of how our risk assessments are performed.

> ***Reviewer 1:***
> *3- How was the proportion of users of symptom checkers based on time and geography? in case of the balanced data availability, the result can be promising and we can compare the results for users with the same country. We need to make sure that the sampling has been done correctly with enough cases for each country as we use the*

data of positive cases according to the country (John's Hopkin's Uni data). More clarification in methodology is really required. We need to make sure that we have had population in both variables with enough numbers in each cluster in which each cluster is for a country.

We thank the reviewer for their concerns. Since Symptoma and to our knowledge any other symptom checker still do not have the same reach as a country-wide testing strategy, unbalanced data was always expected. We filtered out countries, which do not show consecutive data in our time frame:

Only countries with consecutive data, that being no days without a single high-risk assessment given, between 7th of April and 1st of November 2020 were included in the further analysis.

Additionally, we scaled the data to adjust for different magnitudes:

Additionally, we scaled the data so that zero equals the minimum and one equals the maximum. This was done to make the time-series comparable visually and to protect the sensitive user numbers.

Reviewer 1:
4- in discussion, we need to justify this controversy that the positive cases in each country are those whose PCR test is positive, However, the symptom checkers might be those who are positive of COVID 19, but they did not do the PCR test. Thus, the study population of the two variables might be different.

We strongly agree that there are underlying key differences in the populations reflected by the two data sources used within our manuscript. For example, there are clear weekly seasonality patterns in the data provided by Johns Hopkins. Our preprocessing steps, namely, applying a smoothing kernel and min-max scaling addresses this somewhat. However, we do note that this does not solve potential differences in the sampling population. We have added the following to our discussion to reflect the reviewer's valid concern:

Note that the official COVID-19 case numbers only cover the part of the population that got a positive PCR test result. This excludes people that are infected but did not get tested. Symptom checkers provide a risk assessment based on the information provided by the user. A high risk assessment does not imply a positive test result. This leads to the two populations of our comparison being different.

Reviewer 1:
5- More information regarding the topic is required for discussion to compare the result with the other works and interpret the findings. The discussion and conclusion need update and improvement.

Reviewer 2:
1.The methodology of the paper should be succinctly stated. Like study design, study setting. I do not see any details for so.

We adjusted the abstract, introduction and objectives to reflect our methodology more succinctly. Our objectives now state:

Objectives
Assess the correlation between COVID-19 risk assessments from an online symptom checker and current trends in COVID-19 infections and investigate the relationship between those correlations and four quality of life measures.

Further, we added the following section to the end of the introduction to provide an additional overview.

With regards to the COVID-19 pandemic, we investigate whether the lab-confirmed numbers of new COVID-19 cases correlate with the number of high COVID-19 risk assessments given by the 'Symptoma' symptom checker. Similar correlation analysis were performed by others in the context of temperature [7,8], air pollution [9], and income inequality [10]. We show that our hypothesis holds for a majority of countries, the count of high-risk assessments reflecting the trends in COVID-19 cases nationally. Further, we relate these country-wise correlations with 4 different socio-economic factors and find a significant connection to self-reported health from the OECD Better Life Index 2017. Lastly, we investigate the optimal lag between the official case counts and the symptom checker assessments to maximise correlations to indicate whether the assessments could be predictive of the pandemic progression.

Reviewer 2:
2.I see that authors emphasized in the the abstract and write up on digital solutions, but talk briefly on the digital health approaches .

We thank the reviewer for the input. We expanded upon our previous introduction to give a broader range of application context and examples of digital health solutions:

Other surveys list the applications as screening, triage, diagnosis, monitoring and contact tracing [4]. A prominent example of contact tracing software is the mobile app TraceTogether used in Singapore [5] to track past contacts of infected individuals. Surveillance as the continuous, systematic collection, analysis and interpretation of health-related data needed for the planning, implementation, and evaluation of public health practice [4] is the goal of Online surveillance-mapping tools like SORMAS[6] and HealthMap[7]. Monitoring systems are usually deployed in a more private context, to

record the condition of already infected individuals. The tool Sense FollowUp app[8] has been used to monitor Ebola patients remotely and raise an alarm in case of increased temperature readings. One specific type of application in the context of triage is online symptom checkers which are experiencing an increase in popularity [9].

Reviewer 2:
3.The objectives stated do not deem aligned with the title, methods and overall manuscript , would suggest revisiting the objectives.

We agree that the focus of the paper was not reflected accurately by the objectives. To address this, the title "Predicting global trends in COVID-19 cases with online symptom checker self-assessments" has been revised to "Correlating global trends in COVID-19 cases with online symptom checker self-assessments" to better reflect our methodology. Likewise, the objectives were changed to:

Assess the correlation between COVID-19 risk assessments from an online symptom checker and current trends in COVID-19 infections and investigate the relationship between those correlations and four quality of life measures.

which reflects better the investigation of the correlation of symptom checker user assessments and global COVID-19 cases.

Reviewer 2:
4.Very little is explained in the section of analysis, I miss a logical sequence. As mentioned, the concepts do not seem to be aligned. This can be made more smooth and reader understandable.

We improved our methods and discussion section to improve the logical sequence. More insight was given to show how the data was generated and underlying goals. The list of countries included in our data set got added to the data section:

This results in a dataset of 18 countries with 208 days in each. The respective countries are: Australia, Austria, Belgium, Canada, Czech Republic, France, Germany, Greece, Hungary, Ireland, Italy, Netherlands, Spain, Sweden, Switzerland, Turkey, United Kingdom, United States. Within the text, we refer to this data as 'Symptoma risk assessments'.

Pre-processing
Delayed reporting of new COVID-19 cases is a well-known problem [12]. To account for this problem and remove unnatural spikes (e.g., weekends), we applied a seven-day mean filter to the COVID-19 cases as well as the Symptoma risk assessment. Additionally, we scaled the data so that zero equals the minimum and one equals the

maximum. This was done to make the time-series comparable visually and to protect the sensitive user numbers.

Additionally, our discussion has been expanded to include the quality of life measures suggested in another comment below.

On the y-axis of Figure 1C-F, we show in addition the QOL measures GINI-Index, self-reported health, life satisfaction, and the percentage of Internet usage for health-related topics. Hence, Figure 1C-F is a scatter plot between those four QOL measures and 'SRCCs between new COVID-19 positive cases and Symptoma high-risk cases'. A linear model was fit to the data of each Subfigure 1C-F and a regression line was drawn to illustrate the result.
We theorized a relationship between health-related usage of the internet SRCC, because people who search for health-related information on the internet are likely to interested in symptom checker results as well. Figure 1E shows no correlation between health-related usage and SRCC. The regression line reflects that with a slope close to 0 (-0.159) and an R^2 of 0.155.
Our reason for choosing the GINI-Index was that the low-income population should be more inclined to use free online services. Thus we theorize that countries with higher inequality would have a larger portion of the low-income population, which would be more inclined to use Symptoma. Figures 1C and 1F show a weak inverse correlation between SRCC and the QOL measures GINI-Index (SRCC: -0.331) and life satisfaction (SRCC: -0.378). The respective p-values (0.084 and 0.779) provided by the linear model show no statistical significance for this relationship. Figure 1D shows a strong negative correlation between SRCC and self-reported health (SRCC: -0.665). The result is supported by the linear model (R^2: 0.443, p-value: 0.003) and is statistically significant.

Lastly, we streamlined our discussion of the correlation shift analysis to better explain the setting and goals of this analysis:

Correlation shift
In the previous section, we showed that there is good agreement between new COVID-19 cases and Symptoma risk assessment for most countries. This yields the question of which variable is time-wise ahead of the other. Do more new COVID-19 cases lead to more persons assessed as high risk by Symptoma or are already more persons assessed as high risk by Symptoma before the official COVID-19 cases increase? To shed light on this question we performed a lagged-correlation analysis. In Figure 2, we show a scatter plot highlighting 12 countries whose optimal shift falls in the closed interval [-15, 15]. We chose this constraint to remove 6 countries whose best shift was directly at the edge of our window of [-25, 25]. For the majority of countries (n: 18) the optimal shift is positive (median: +0.5 days overall 18 countries). The same holds for the filtered set of countries (median: +3 days overall 12 countries). This means that on average the number of users assessed by Symptoma to have a high risk of being infected with COVID-19 increases/decreases before the official number of cases

increases/decreases. By introducing shifts, the mean SRCC increases from 0.342 (median: 0.353) to 0.411 (median: 0.419).

Reviewer 2:

5.The authors should give a sound rationale to explain the link between the type of analyses and a justification for doing such analysis. In my opinion there could have been more robust analysis(regression)to do so.

We thank the reviewer for their comment. The key analysis of the manuscript is the correlation shift analysis. We hypothesised that this analysis would give insight as to whether user-interaction with a symptom checker can reflect a pandemic's progression. Should this be the case, and as commented on within the manuscript, then further work invested into predictive modelling and forecasting would be planned. As mentioned above, we have added additional text to both the Abstract, Introduction and Methods to help clarify our objectives.

Reviewer 2:

6. I would suggest that the authors also categorize the countries region/ income status wise and then explain the results

We followed the reviewer's suggestion for a great addition to our manuscript. We selected four relevant Quality of Life (QoL) metrics and tested them for association with our previous results. Specifically, from the OECD Better Life Index 2017 (BLI 2017) we selected "self-reported health" and "life satisfaction". the GINI Index, which represents income inequality, and, lastly, the "percentage of internet users seeking health information". We assessed the relationship of those measures with the correlation between Symptoma users assessed with a high risk for COVID-19 and the global COVID-19 cases provided by Johns Hopkins University. We found a significant negative relationship between self-reported health and the correlation between Symptoma and global COVID-19 cases (SRCC: -0.665, R^2: 0.443, adj. p-value: 0.003).

Reviewer 2:

figures are very hap hazard and confusing, have a lot more room for improvement

We are sorry to hear that the presentation of data was confusing. In light of this comment, we have made the following figure alterations. Figure 1 was changed to show the normalized time series of the Symptoma assessment and the COVID-19 cases for a few example country in subfigures A & B. In subfigures C-F, the relationship between the above QoL measures and the calculated SRCC between Symptoma and new COVID-19 cases is given. Figure 2 was reworked entirely to remove unnecessary information; now only the core information of the shifted correlation analysis is shown. Namely, only the optimal lag per country is presented.

Further, the countries at the edge of our shift window of [-25, 25] were removed. We believe that with all of the above alterations, figure clarity is greatly enhanced.

Lastly, we would again like to thank the reviewers for their comments. As outlined above, we have greatly improved the manuscript based on their feedback.

Thank you for your time and consideration.

Yours Sincerely,
Marc Zobel
Alistair Martin
Jama Nateqi
Bernhard Knapp
