# Supplementary material for: Correlating global trends in COVID-19 cases with online symptom checker self-assessments
Source: PLoS One. 2023 Feb 10;18(2):e0281709. doi: 10.1371/journal.pone.0281709 (PMC9917242; doi:10.1371/journal.pone.0281709)
Supplement: Attachment — Submitted filename: Rebuttal Letter.pdf [file pone.0281709.s003.pdf]

**PLOS ONE (minor) Revision**

Dear Reviewers,

On behalf of all the authors of the paper, we would like to thank you for the in-depth review. We have implemented most of the suggested changes after much careful consideration. In the following sections, we describe in detail how we addressed each comment:

Methods:

*lack of the section to introduce the data: Thanks for enhancing this section. It would be beneficial to provide the full data set as supplement file or refer to an externally available repository that contains the data. Still missing is the absolute numbers of each data set.*

We have expanded the Data Availability statement to include a repository link in which the scaled user count per country per day over the period analysed is stored. Further, we provide all other data (e.g., QoL metrics) used within our manuscript for complete reproducibility. We feel strongly that this meets the Minimal Data Set Definition outlined by PLOS; namely, it allows the reproduction of all results and figures. In addition to the above, the Data Availability statement also outlines the process by which a reader can obtain the underlying untransformed data, i.e., the number of users assessed as "high risk" per day for each country included in this study. Specifically, the reader must request this data via email. For the review process, we are happy to supply the data via the editor to the reviewer, should they so wish.

*Especially for the Quality of life measures from the OECD a detailed description is missing. Also descriptive numbers for all data sets used for the paper are required to fully understand it. It is important to see the 4 QoL metrics per country in detail.*

We have expanded the relevant section within Materials & Methods to detail how each Quality of Life (QoL) metric is derived. Furthermore, the descriptive summary statistics on these QoL metrics globally, e.g., the mean and standard deviation, are now provided to allow for context outside the subset of countries featured in our study. Lastly, in the supplementary information, we now give the exact numbers for the four QoL metrics for the countries featured in our study.

*the correlation analysis description needs further rework, since it is still not comprehensible how you exactly did that "lagged-correlation" analysis.*

We thank the reviewer for their comment. We have expanded the correlation analysis section to clarify the lagged correlation analysis. Specifically, we now provide a formula such that it is defined explicitly which should prevent future uncertainty.

*It remains unclear why the authors team has chosen Germany and US to include in Figure 1.*

Within the text, we stated that these countries are merely examples, one for which there is a high correlation (Germany) and one for which there is a low correlation (USA). We felt that an example of the two data sources overlapped provided understanding of the work. However, we wished not to be accused of cherry-picking by featuring only a good example; thus, we displayed the two countries mentioned above.

However, and likely the source of the reviewer's comment, the explanation regarding this selection, and the associated discussion, was separate from the first introduction of Figure 1. We suggest that this separation led to the reviewer's confusion. The relevant text has been shifted such that we give the context for the country selection alongside the Figure's first introduction.

*Also it would be good to not only introduce the mean/median correlation of all included countries but instead add it for each country (e.g. in a table)*

We have added the requested table to the supplementary information and included reference to it within the manuscript.

Results + Discussion:

*This section mixes up results and discussion. You need to strictly divide between the results based on your methods and then discuss them. Currently, you mix results and discussion.* Also, the sequence is still confusing here. Still needs rework here.

We apologise for the lack of clarity here in our previous manuscript. In light of this comment, we implemented a significant restructuring of the manuscript to make a clear distinction between our results and their discussion. This restructuring includes introducing a dedicated discussion section whereby we comment upon the results and what may be driving said observations.

*Confidence interval is missing for the regression line.*

We have now added confidence intervals for all figures containing regressions (Figures 1C-1F). Please note that we had already reported significance values (p-values) for these models within the text to indicate the goodness of fit.

*Correlation shift results section needs further improvement. The correlation improvement by country is very small and without the absolute value of the correlation for each country without the shift not useful.*

As requested above, we have added a table containing the correlation for each country as a table within the supplementary information. Further, this table also includes the maximum correlation obtained when we apply a shift and the shift with which this occurs. We reference this table within the text, and the information is used throughout the discussion.

*Conspicuous is the largest increase of 0.103 for Spain, that you unfortunately did not take into account for any discussion.*

We thank the reviewer for this observation. After investigation, we added the following discussion to the manuscript:

"While the correlations increased universally with the introduction of a shift, for some countries, the optimal shift was negative. For example, for Spain, which exhibited the largest increase in correlation (+0.103), the optimal shift was -12 days, i.e., the COVID-19 pandemic progression precedes the trends observed in the chatbot. We believe this is due to the number of recorded cases in Spain being almost monotonic over the period analyzed. Other countries experience an initial peak followed by a Summer season with fewer infections. For Spain, infection numbers were instead stable after the initial onset. The absence of an initial peak was likely due to limited testing capacity when Spain was one of the epicentres of the pandemic's first wave in Europe [27]."

*Figures: still need rework, Figure 1 (A) + (B): x-axis labels missing,*

We have added labels to Figure 1B. As Figure 1A shares an x-axis with Figure 1B, we have chosen to drop the axis labels as the elements do not add new information to the graphic.

*Figure 1 (C) - (F): x-axis labels + scale is not explained before in detail,*

As suggested by the reviewer above, we added further explanation of these metrics to our Materials & Methods section, including a complete description of the respective scales. To aid the reader, we have also briefly reiterated what each metric represents, when the name is unclear, within the Results & Discussion.

*you also decided to cut x-axis e.g. GINI score was introduced from 0 - 1 but not reflected in the Figure.*

Plotting the complete range of the GINI score and other QoL metrics is unreasonable, considering that the subset of countries featured in our study appears only in a small region. Including everything would lead to an uninformative visualisation; the data is forced together, even overlapping. However, we understand that limiting the axis to that of the subset could be used to present a false narrative. We prevent this situation by supplying the regression for each metric against the correlation, including the significance, which objectively indicates the strength of relationships to the reader.

*You mention the 4 correlations 0 or smaller in the text for GINI but not this other plots (D), (E), (F)*

We are unsure about the reviewer's comment as the previous iteration of our manuscript did not contain a discussion about the subset of correlations that were negative and how they related to any of the QoL metrics, including the Gini index. The negative correlations and potential reasons why the respective countries diverged from the chatbot trends were discussed in detail but independently from the QoL metric analysis. Regardless, we hope that the extensive revision of the results and discussion section, as mentioned above, has provided additional clarity to this section and resolved the review's concern.

In addition to the changes based on the reviewer's comments, we want to draw attention to a slight correction we made to the manuscript regarding our data pipeline. We stated previously that we filtered countries which lacked data for even a single day. This was incorrect. Instead, we filtered countries for which less than 200 of the 208 days had data, i.e., more than eight days missing. For reference, eight days is less than 4% of the analysed time period. Zeros were inserted for days without values. Please note that this had no downstream effect, as the previous manuscript versions were already based on this filter. Further, during this review, the manuscript underwent another editing process which resulted in a couple of small changes. The last notable change is that the author list has been reordered to reflect changes within our company over the revision period. We hope this change can be propagated in your system and is not a point of concern.

Lastly, we would again like to thank the reviewers for their comments. As outlined above, we have greatly improved the manuscript based on their feedback.

Thank you for your time and consideration.

Yours Sincerely,

Marc Zobel

Bernhard Knapp

Jama Nateqi

Alistair Martin
